# Endogenous PGE_2_ induces MCP-1 expression via EP4/p38 MAPK signaling in melanoma

**DOI:** 10.3892/ol.2012.1047

**Published:** 2012-11-27

**Authors:** MINGRUI TANG, YUXIN WANG, SIHUAN HAN, SHU GUO, NAN XU, JIAYAN GUO

**Affiliations:** Department of Plastic Surgery, First Hospital of China Medical University, Liaoning 110001, P.R. China

**Keywords:** prostaglandin E_2_, cyclooxygenase-2, macrophage chemoattractant protein 1

## Abstract

It has been demonstrated that cyclooxygenase-2 (COX-2) is expressed in melanoma tissues and prostaglandin E_2_ (PGE_2_) is produced by melanoma cells *in vitro*. However, the roles of COX-2/PGE_2_ in melanoma are largely unknown. In the present study, we set out to analyze the correlation of endogenous PGE_2_ with the expression of macrophage chemoattractant protein-1 (MCP-1) and to identify the signaling pathway involved. It was found that MCP-1 mRNA was heterogeneously expressed in 18 melanoma tissue specimens, and the levels of MCP-1 mRNA were positively correlated with those of COX-2 mRNA. Inhibition of endogenous PGE_2_ production by a COX-2 inhibitor, COX-2 siRNA or an NFκB inhibitor suppressed MCP-1 expression, whereas treatment with TNF-α (to stimulate endogenous PGE_2_ production) or exogenous PGE_2_ enhanced MCP-1 expression in melanoma cells. Both the EP4 antagonist and the p38 MAPK inhibitor reduced MCP-1 production in melanoma cells, and abrogated the increased MCP-1 secretion induced by TNF-α or exogenous PGE_2_. Conditioned medium from melanoma cells promoted macrophage migration, which was blocked by inhibitors of the PGE_2_/EP4/p38 MAPK signaling pathway. These results indicate that endogenous PGE_2_ induces MCP-1 expression via EP4/p38 MAPK signaling in an autocrinal manner in melanoma, and melanoma cell-derived PGE_2_ may be involved in macrophage recruitment in the melanoma microenvironment.

## Introduction

It is widely accepted that prostaglandin E_2_ (PGE_2_) is important in the pathogenesis of tumors. Early evidence was indirectly provided by epidemiological and laboratory studies, which revealed that cyclooxygenase-2 (COX-2) inhibitors are capable of suppressing the incidence of colorectal and breast cancer (1–3). By contrast, COX-2 overexpression was observed in a series of types of human cancer (4–6), while mice with COX-2 overexpression were found to be more susceptible to carcinogenesis (7). As a key enzyme in the synthesis of PGE_2_, COX-2 is considered to act via PGE_2_. Certain studies have directly shown that PGE_2_ is involved in several crucial aspects of malignant tumors, including proliferation, invasion, migration and angiogenesis (8–11).

COX-2 expression has also been demonstrated to be present in human melanoma tissue, whereas it has been revealed to be absent in benign melanocytic nevi and normal epithelium (12). Certain melanoma cell lines tested were found to produce PGE_2_, which was suppressed by NS398, a specific inhibitor of COX-2 (12). The roles of PGE_2_ in melanoma have not been extensively investigated. PGE_2_ suppression has been demonstrated to have no effects on melanoma cell proliferation, but to reduce cell invasion, indicating that PGE_2_ may be implicated in melanoma progression (12). Recently, several studies from one laboratory provided further evidence for the involvement of PGE_2_ in melanoma metastasis. It was demonstrated that green tea catechin, grape seed proanthocyanidins and berberine are capable of suppressing melanoma cell invasion and migration by inhibition of COX-2, PGE_2_ and PGE_2_ receptors (13–15).

In the present study, we identified a novel role of PGE_2_ in melanoma. We found that via EP4/p38 MAPK signaling, endogenous PGE_2_ upregulates the expression of macrophage chemoattractant protein-1 (MCP-1), an important chemoattractant to macrophages, suggesting that PGE_2_ may be involved in macrophage recruitment in melanoma. Macrophage infiltration is often present in melanoma tissues, and studies have revealed that macrophages facilitate melanoma angiogenesis (16) and invasion (17). However, MCP-1 is also able to recruit natural killer (NK) cells and cytotoxic lymphocytes (CTLs), which exert an inhibitory effect on melanoma cells (18). Overall, these data imply that PGE_2_ may play more complex roles in melanoma than previously known.

## Materials and methods

### Melanoma cells and specimens

Melanoma cell lines, A2058 and MeWo, were purchased from the American Type Culture Collection (ATCC; Manassas, VA, USA) and were cultured in RPMI-1640 medium supplemented with 10% fetal bovine serum, 100 mg/ml penicillin and 100 mg/ml streptomycin at 37°C in an incubator with 5% CO_2_. Eighteen melanoma specimens were obtained with consent from patients undergoing surgery at the First Hospital of China Medical University (Liaoning, China). This study was approved by the Ethics Committee of China Medical University.

### Real-time reverse transcription PCR

Total RNA was isolated from cells and tissues by TRIzol (Takara, Dalian, China) and reverse transcribed by the PrimeScript RT Reagent kit (Takara) according to the manufacturer’s instructions. Primer sequences for COX-2 and MCP-1 were as described in a previous study (19). Real-time PCR was performed using SYBR Premix Ex *Taq* II (Takara) in an Applied Biosystems 7500 Fast Real-Time PCR system (Applied Biosystems/Life Technologies Corporation; Carlsbad, CA, USA). The housekeeping gene GAPDH was used as an internal control. Gene expression was quantified by the comparative CT method, normalizing CT values of the target gene to those of GAPDH and calculating relative expression values.

### Western blot analysis

Cells were lysed with sample buffer containing 50 mmol/l Tris-HCl (pH 6.8), 100 mmol/l dithiothreitol (DTT), 2% SDS, 0.1% bromophenol blue and 10% glycerol. Protein (20 *μ*g) was separated in a 10% sodium dodecyl sulfate (SDS)/acrylamide gel and was then transferred to a nitrocellulose membrane, which was blocked at 4°C in phosphate-buffered saline (PBS) supplemented with 0.1% Tween and 10% milk powder. The membrane was then incubated with the primary antibody (1:1,000) at 4°C overnight. After the membrane was washed three times with PBS, the corresponding second antibody was added (1:2000) for 1 h at room temperature. Primary antibodies for COX-2, EP4, phosphorylated p38 MAPK, β-actin and the corresponding secondary antibodies were purchased from Santa Cruz Biotechnology, Inc. (Santa Cruz, CA, USA). The human β-actin gene was used as an internal control.

### Enzyme-linked immunosorbant asssay (ELISA)

Concentrations of PGE_2_ and MCP-1 in cell culture supernatants (2×10^5^/well) were measured using Quantikine ELISA kits (Boster Biological Technology, Ltd.; Wuhan, China) according to the manufacturer’s instructions. The detection limit of the assay was 4 pg/ml.

### RNA interference

The COX-2 siRNA and nonsilencing control siRNA plasmids were provided by Takara. Cells were seeded into a 24-well plate at a density of 5×10^5^/well for 24 h, and were then transfected with siRNA plasmids using Lipofectamine 2000 (Invitrogen Life Technologies; Carlsbad, CA, USA) according to the manufacturer’s instructions. Silencing of COX-2 expression in cells following transfection was verified by western blot analysis.

### Macrophage migration assay

Macrophages (2×10^5^) were added to the upper chamber of a transwell insert with an 8-*μ*m pore size membrane. Conditioned medium from melanoma cells was added to the lower chamber. Cells were allowed to migrate for 6 h at 37°C and 5% CO_2_, then fixed and stained with Diff-Quick stain (International Reagents Corp., Kobe, Japan) according to the manufacturer’s recommendations. The number of migrated cells in five random microscopy fields per well were counted. Experiments were performed in triplicate.

### Statistical analyses

A Spearman’s rank correlation was used to analyze the correlation between MCP-1 and COX-2 expression in melanoma specimens. Differences in the protein content of the cell culture supernatant, the cell mRNA level and macrophage migration were evaluated using a Student’s t-test or a one-way analysis of variance (ANOVA). Statistical analyses were conducted using the Statistical Package for the Social Sciences (SPSS) software, version 13.0 (SPSS Inc.; Chicago, IL, USA). P<0.05 was considered to indicate a statistically significant difference.

## Results

### MCP-1 expression is correlated with COX-2 expression in melanoma tissue

**Figure 1.**
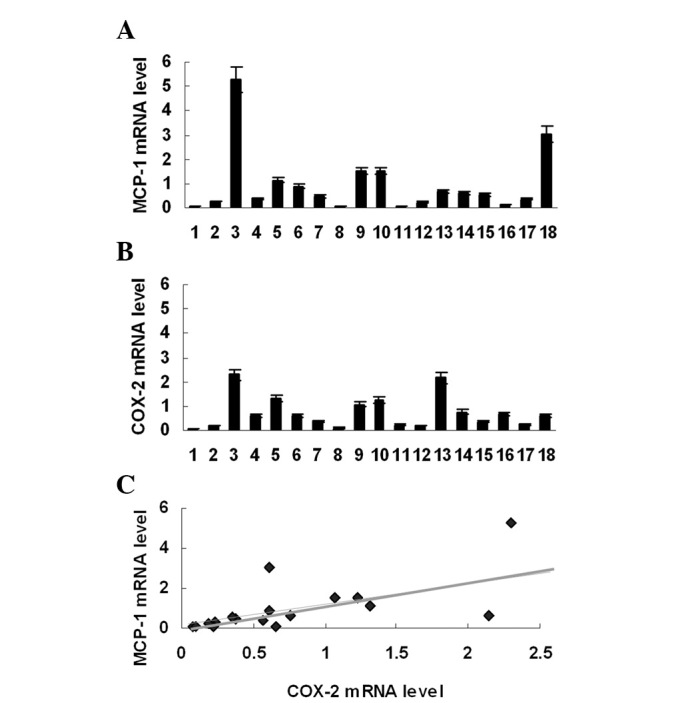
MCP-1 expression is correlated with COX-2 expression in melanoma specimens. (A) MCP-1 and (B) COX-2 mRNA expression detected in 18 melanoma specimens by real-time PCR. (C) Expression levels of COX-2 mRNA are positively correlated with those of MCP-1 mRNA in melanoma specimens; P<0.01. MCP-1, macrophage chemoattractant protein-1; COX-2, cyclooxygenase-2.

MCP-1 mRNA expression was examined in 18 melanoma specimens. Real-time PCR revealed that all specimens expressed MCP-1 mRNA (Fig. 1A). However, MCP-1 mRNA expression in melanoma was heterogeneous; the highest mRNA expression level was >100-fold greater than the lowest. In order to analyze the correlation of MCP-1 expression with PGE_2_ production, COX-2 mRNA expression was subsequently investigated in the same group of melanoma specimens. It was found that COX-2 mRNA was also expressed in these specimens (Fig. 1B) and that the levels of COX-2 mRNA were positively correlated with those of MCP-1 mRNA (Fig. 1C).

### PGE_2_ production is dependent on COX-2 expression and NFκB activation in melanoma cells

**Figure 2.**
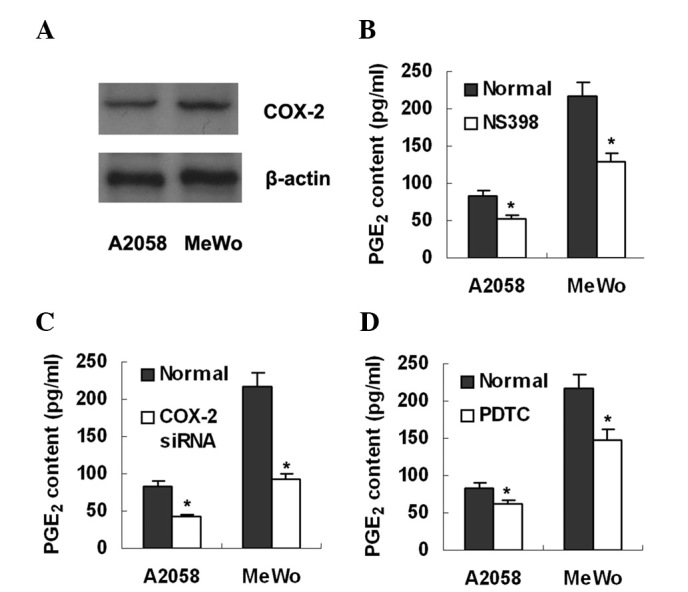
PGE_2_ production is dependent on COX-2 expression and NFκB activation in melanoma cells. (A) COX-2 expression is shown in A2058 and MeWo melanoma cells by western blot analysis. (B–D) ELISA detection demonstrates that both melanoma cell lines secrete the PGE_2_ protein, which is reduced by (B) the COX-2 inhibitor NS398, (C) COX-2 siRNA and (D) the NFκB inhibitor PDTC. ^*^P<0.01 vs. normal. PGE_2_, prostaglandin E_2_; COX-2, cyclooxygenase-2; ELISA, enzyme-linked immunosorbent assay.

Western blot analysis and ELISA were used to detect COX-2 expression and PGE_2_ production, respectively, in two melanoma cell lines, A2058 and MeWo. The results showed that both cell lines expressed COX-2 protein (Fig. 2A) and secreted PGE_2_ into culture supernatant at 84 and 216 pg/ml for A2508 and MeWo cell lines, respectively (Fig. 2). To further explore whether the production of endogenous PGE_2_ is dependent on COX-2 expression, the cell lines were treated with a COX-2 inhibitor, NS398 (Sigma; St Louis, MO, USA; 100 *μ*mol/l). ELISA detection revealed that the PGE_2_ levels in the culture supernatant had decreased in the two cell lines (Fig. 2B). Similarly, treatment with COX-2 siRNA was also demonstrated to reduce PGE_2_ production in the two cell lines (Fig. 2C). To determine whether NFκB is involved in PGE_2_ production, we treated the melanoma cells with an NFκB inhibitor, PDTC (Sigma; 100 *μ*mol/l). It was observed that PGE_2_ production was significantly inhibited (Fig. 2D).

### MCP-1 expression is downregulated by inhibition of endogenous PGE_2_ production

**Figure 3.**
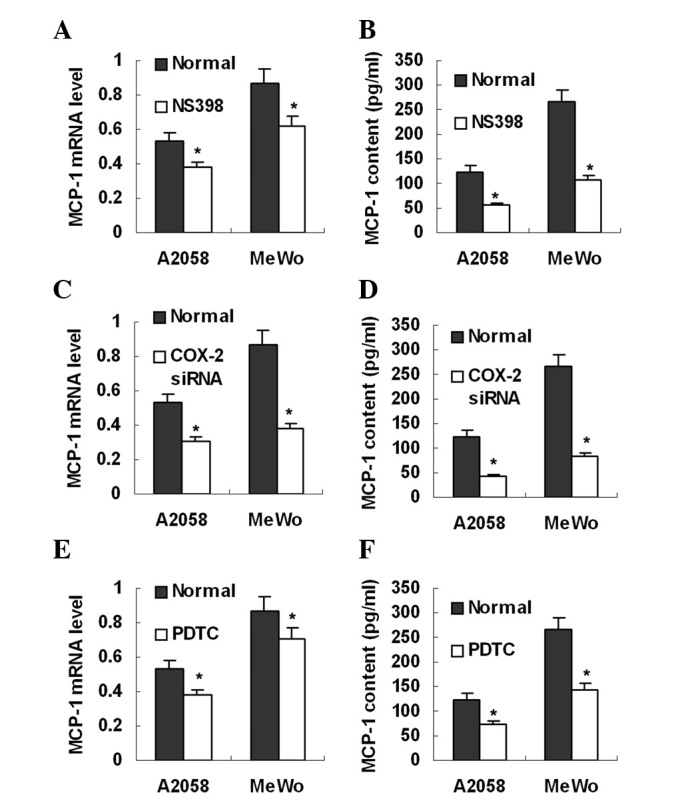
MCP-1 expression is downregulated by inhibition of endogenous PGE_2_ production. (A, C and E) MCP-1 mRNA levels are shown to decrease after melanoma cells are treated with NS398, COX-2 siRNA and PDTC, respectively. (B, D and F) NS398, COX-2 siRNA and PDTC inhibit secretion of MCP-1 protein into the melanoma cell culture supernatant. ^*^P<0.01 vs. normal. MCP-1, macrophage chemoattractant protein-1; COX-2, cyclooxygenase-2; PGE_2_, prostaglandin E_2_.

Real-time PCR and ELISA revealed that the two melanoma cell lines expressed MCP-1 mRNA (Fig. 3) and secreted MCP-1 protein (Fig. 3), 124 pg/ml for A2058 and 266 pg/ml for MeWo, into the culture supernatant. To investigate whether endogenous PGE_2_ is involved in MCP-1 expression, the melanoma cell lines were treated with NS398 and COX-2 siRNA, respectively. It was found that treatment with either NS398 or COX-2 siRNA markedly inhibited MCP-1 mRNA expression and protein production in the melanoma cell lines (Fig. 3A–D). In addition, MCP-1 expression in these cell lines was also reduced by the NFκB inhibitor, PDTC (Fig. 3E and F). These data indicate that inhibition of endogenous PGE_2_ production suppresses MCP-1 expression in melanoma cells.

### MCP-1 expression is upregulated by endogenous and exogenous PGE_2_

**Figure 4.**
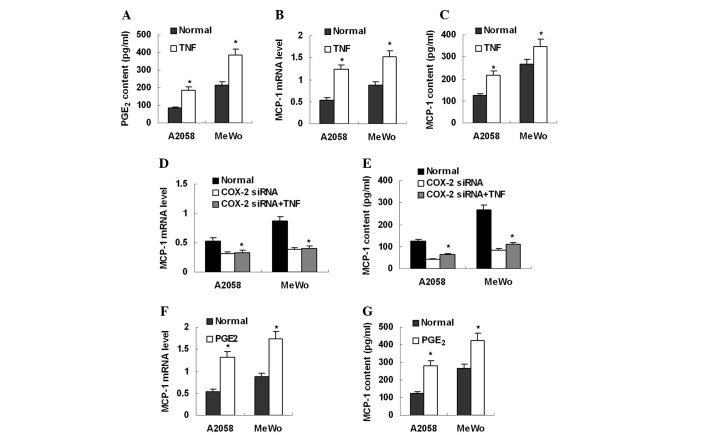
MCP-1 expression is upregulated by endogenous and exogenous PGE_2_. (A) Exogenous TNF-α enhances PGE_2_ secretion in melanoma cell lines. (B and C) Exogenous TNF-α stimulates MCP-1 mRNA expression and protein secretion in melanoma cell lines. (D and E) TNF-α treatment is not able to stimulate MCP-1 mRNA expression and protein production in melanoma cells pre-treated with COX-2 siRNA. (F and G) Exogenous PGE_2_ promoted MCP-1 mRNA expression and protein secretion in melanoma cells. ^*^P<0.01 vs. normal. MCP-1, macrophage chemoattractant protein-1; PGE_2_, prostaglandin E_2_.

To stimulate endogenous PGE_2_ production, mealnoma cell lines were treated with TNF-α (Sigma; 100 ng/ml), a strong PGE_2_ inducer. As expected, TNF-α treatment significantly increased PGE_2_ production (Fig. 4A). MCP-1 expression was simultaneously elevated in these cells (Fig. 4B and C). However, TNF-α treatment was not able to stimulate MCP-1 expression in melanoma cells pre-treated with COX-2 siRNA (Fig. 4D and E). These data suggest that PGE_2_ mediates TNF-α-induced MCP-1 upregulation. Furthermore, it was investigated whether exogenous PGE_2_ was capable of enhancing MCP-1 expression in melanoma cells. After treatment with exogenous PGE_2_ (Sigma; 100 *μ*mol/l) for 8 h, melanoma cells were found to express more MCP-1 mRNA and secreted more MCP-1 protein into culture supernatant (Fig. 4F and G).

### EP4/p38 MAPK signaling is involved in MCP-1 upregulation by PGE_2_

**Figure 5.**
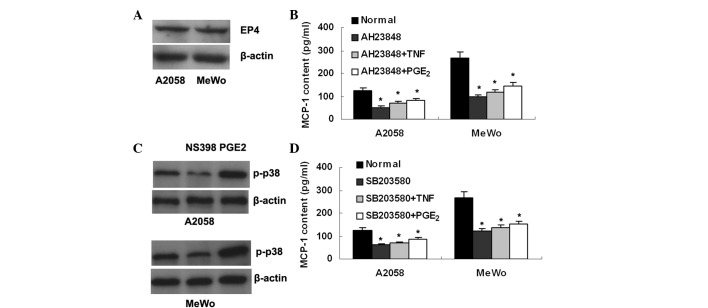
EP4/p38 MAPK signaling is involved in MCP-1 upregulation by PGE_2_. (A) The PGE_2_ receptor, EP4, is shown in A2058 and MeWo melanoma cells by western blot analysis. (B) The EP4 antagonist, AH23848, reduces MCP-1 production in melanoma cells, and blocks the increased MCP-1 production induced by TNF-α or exogenous PGE_2_. (C) Expression of p-p38 MAPK is suppressed by NS398, whereas it is enhanced by exogenous PGE_2_ in the melanoma A2058 and MeWo cells. (D) The p38 MAPK inhibitor, SB203580, decreases MCP-1 production in melanoma cells and abrogates the increased MCP-1 production induced by TNF-α or exogenous PGE_2_. ^*^P<0.01 vs. normal. MCP-1, macrophage chemoattractant protein-1; PGE_2_, prostaglandin E_2_.

The PGE_2_ receptor EP4 was observed to be expressed in the two melanoma cell lines by western blot analysis (Fig. 5A). To further analyze the role of EP4 in the PGE_2_-MCP-1 axis, the cell lines were treated with an EP4 antagonist, AH23848 (100 *μ*mol/l). ELISA detection revealed that MCP-1 production was significantly inhibited by AH23848 (Fig. 5B). In addition, AH23848 blocked the induction of MCP-1 caused by TNF-α or exogenous PGE_2_ (Fig. 5B). Overall, these data suggest that PGE_2_ stimulates MCP-1 expression via EP4 in an autocrinal or paracrinal manner.

Next we attempted to identify the intracellular molecule mediating PGE_2_/EP4 signaling in the upregulation of MCP-1. It was found that expression of phosphorylated p38 MAPK (p-p38) was suppressed by NS398, whereas it was enhanced by exogenous PGE_2_, in mealnoma cells (Fig. 5C). Furthermore, the p38 MAPK inhibitor SB203580 was revealed to suppress MCP-1 production in melanoma cells, and as with AH23848, SB203580 abrogated MCP-1 upregulation caused by TNF-α or exogenous PGE_2_ (Fig. 5D). Overall, these data suggest that p38 MAPK may be an intracellular signal molecule linking PGE_2_/EP4 with MCP-1.

### Macrophage migration assays

**Figure 6.**
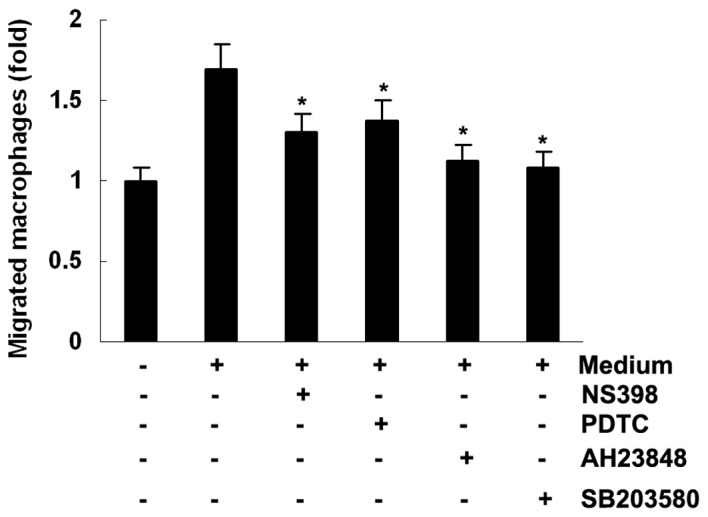
Macrophage migration assays. Conditioned medium from the MeWo melanoma cell line promotes macrophage migration, which is blocked by NS398, PDTC, AH23848 or SB203580. ^*^P<0.01 vs. normal.

To investigate whether MCP-1 expression is associated with macrophage chemotaxis, macrophage migration was analyzed in transwell inserts *in vitro*. On addition of conditioned medium from the MeWo melanoma cell line to the lower chamber, the number of migrated macrophages increased 1.7-fold. However, the increased macrophage migration was suppressed by conditioned medium from melanoma cells treated with NS398, PDTC, AH23848 and SB203580 (Fig. 6).

## Discussion

It has been well established that the normal level of PGE_2_ is indispensable to the maintenance of the functional integrity of the gastrointestinal tract, kidney and cardiovascular system, whereas excessive production of PGE_2_ is frequently linked to certain inflammatory conditions, including rheumatoid arthritis and osteoarthritis (20). As a known pro-inflammatory mediator, PGE_2_ is responsible for inflammatory pain and fever, the most common signs of inflammatory disease. However, PGE_2_ also exhibits anti-inflammatory properties, as it has been observed to inhibit the production of inflammatory cytokine TNF-α by macrophages (21,22). In contrast to the potentially dual roles of PGE_2_ in inflammation, PGE_2_ is observed to play a single pro-tumor role in the pathogenesis of human cancer (23).

In the present study, two melanoma cell lines were found to secrete a detectable amount of PGE_2_ into the culture supernatant. Furthermore, our study revealed that PGE_2_ secretion was suppressed by a COX-2 inhibitor or COX-2 siRNA. These data indicate that production of endogenous PGE_2_ in melanoma cells is dependent on COX-2 expression. The mechanisms underlying COX-2 overexpression in melanoma cells are largely unknown. In physiological conditions, COX-2 was not expressed in the majority of tissues. However, COX-2 expression may be induced significantly by certain stimuli, including inflammatory cytokines and growth factors. In the current study, PGE_2_ production was observed to be markedly suppressed by an NFκB inhibitor, suggesting that NFκB activation may play an important role in COX-2 expression as well as in PGE_2_ production in melanoma.

The roles of PGE_2_ in melanoma have not been widely investigated, despite several recent studies demonstrating that PGE_2_ is involved in melanoma invasion and migration (13–15). In this study, several lines of evidence demonstrate that PGE_2_ is a potential inducer of MCP-1 expression in melanoma. Firstly, the level of COX-2 mRNA was positively correlated with that of MCP-1 mRNA in the melanoma specimens. Secondly, MCP-1 expression was downregulated significantly in melanoma cells treated with a COX-2 inhibitor or a COX-2 siRNA plasmid to reduce PGE_2_ production. Thirdly, pre-treatment with a COX-2 inhibitor clearly abrogated TNF-α-induced MCP-1 upregulation in melanoma cells. Finally, MCP-1 expression was elevated in melanoma cells treated with exogenous PGE_2_. The involvement of PGE_2_ in MCP-1 regulation has also been observed in other organs by two recent animal studies. It has been demonstrated that overproduction of PGE_2_ was able to upregulate MCP-1 expression in the stomach (19). However, inhibition of PGE_2_ production by a COX-2 inhibitor or by COX-2 knockdown significantly reduced MCP-1 expression in several organs in mice (24).

There are four G protein-coupled receptors (GPCRs) that bind extracellular PGE_2_ molecules, EP1-4. EP4 is extensively involved in tumor growth, angiogenesis and metastasis (25–27). The intracellular signaling pathway initiated by PGE_2_/EP4 is mainly mediated by cAMP/protein kinase A (PKA) (27). In addition, phosphatidylinositol 3-kinase/protein kinase B (PI3K/PKB) (28) and extracellular-signal regualted kinase (ERK) (29) have been implicated in this process. In melanoma, Vaid *et al* demonstrated that PGE_2_/EP4-ERK signaling is correlated with melanoma invasion and migration (14). In the present study, we revealed that EP4/p38 MAPK signaling is involved in PGE_2_-induced MCP-1 upregulation in an auto-crinal manner, based on the findings that the EP4 antagonist and p38 MAPK inhibitor blocked MCP-1 induction and macrophage migration toward MCP-1. These data suggest that PGE_2_/EP4 may play distinct roles in melanoma via different intracellular signals. Our results are consistent with another study, which indicated that EP4 mediates MCP-1 upregulation and consequent macrophage recruitment in the stomach (19).

The recruitment of tumor-associated macrophages (TAMs) has been recognized as one of the hallmarks of human cancer. TAMs have positive roles in carcinogenesis, via the production of growth factors, cytokines, chemokines and extracellular matrix degrading enzymes. TAMs are derived from circulating monocytes, which are attracted to tumor tissues by chemokines, including MCP-1. As one of the most important macrophage attractants, MCP-1 is overexpressed in a number of types of human cancer (30) and is produced by tumor cells, T lymphocytes, endothelial cells or other stromal cells in the tumor microenvironment (30). However, the mechanisms underlying MCP-1 overexpression in human tumors remain unclear, in particular in melanoma. The present study demonstrated that endogenous PGE_2_ may be an important stimulator for MCP-1 expression, which is speculated to be involved in macrophage infiltration in melanoma. This is due to the *in vitro* finding that MCP-1-conditioned melanoma medium enhanced macrophage migration; however, the increased migration was substantially suppressed when MCP-1 expression in melanoma cells was downregulated by inhibitors of the PGE_2_/EP4/p38 MAPK signaling pathway.

In addition to the pro-tumor role caused by TAMs, MCP-1 is also capable of exerting an inhibitory effect on tumorigenesis, partly via the recruitment of NK cells and CTLs. This is the case with melanoma, as MCP-1 expression has been observed to be associated with the recruitment of CTLs and NK cells *in vitro* and *in vivo*(31). Additionally, animal studies have demonstrated that MCP-1-induced CTL recruitment promotes melanoma cell apoptosis (32), whereas MCP-1 deficiency, which is associated with a decreased infiltration of CTLs and NK cells, enhances melanoma growth and lung metastasis (18). These results indicate that PGE_2_, as an endogenous stimulator for MCP-1, may play dual, or even more complex, roles in melanoma.
